# Supplementary material for: Identification and analysis of genomic regions influencing leaf morpho-physiological traits related to stress responses in greater yam (Dioscorea alata L.)
Source: BMC Plant Biol. 2025 Nov 17;25:1586. doi: 10.1186/s12870-025-07595-3 (PMC12624995; doi:10.1186/s12870-025-07595-3)
Supplement: Supplementary file 1 — Supplementary Material 1. [file 12870_2025_7595_MOESM1_ESM.pdf]

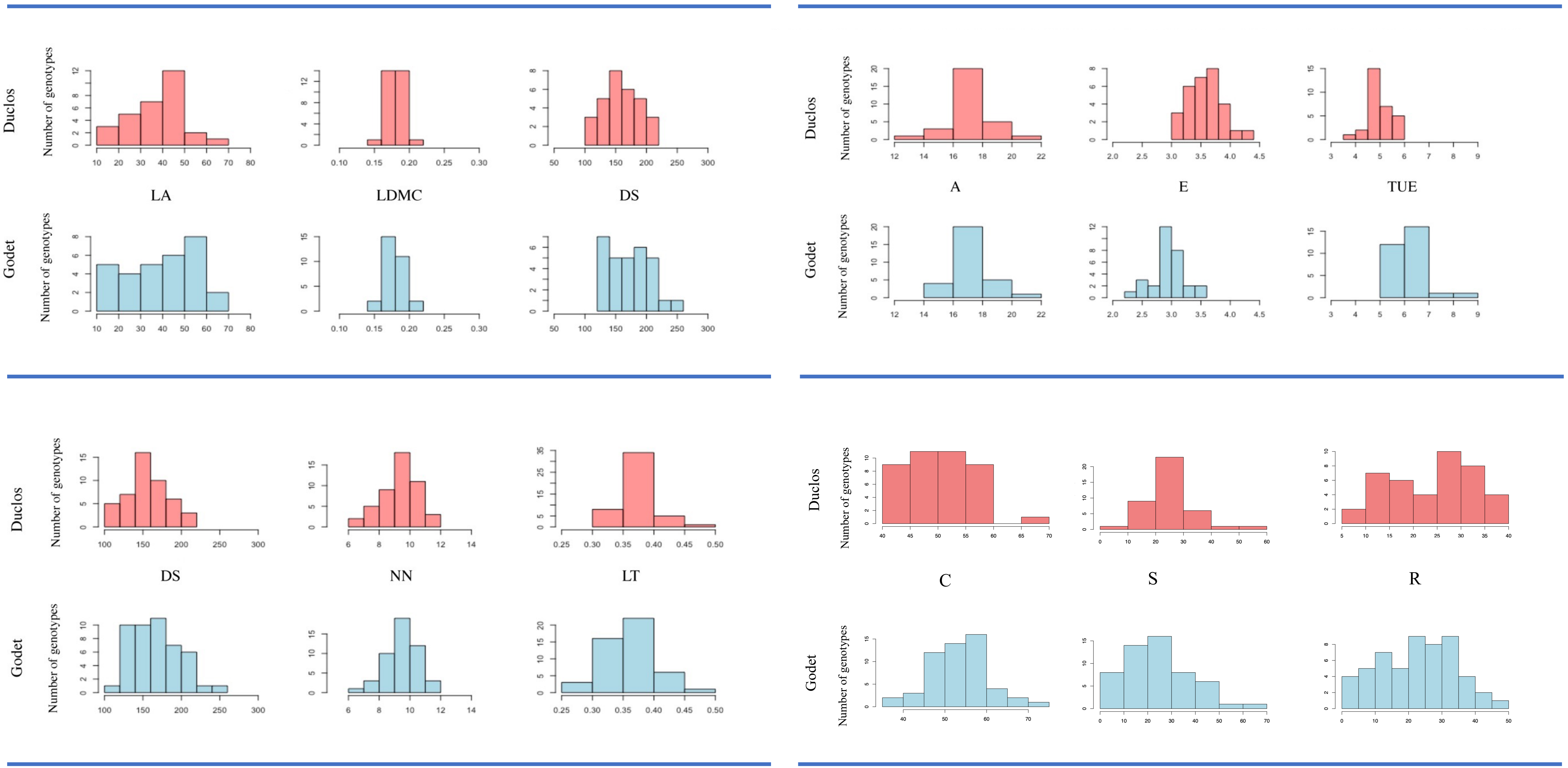

Figure S1. Distribution of each trait under study at two locations, Duclos and Godet. LDMC= leaf dry matter content, LA=leaf area, A=net photosynthesis, E= transpiration rate, TUE= transpiration use efficiency, IS= stomatal index, DS= stomatal density, NN= node number, LT= leaf thickness, C = competitor, S = stress-tolerator, R = ruderal
