# Supplementary material for: Identification and analysis of genomic regions influencing leaf morpho-physiological traits related to stress responses in greater yam (Dioscorea alata L.)
Source: BMC Plant Biol. 2025 Nov 17;25:1586. doi: 10.1186/s12870-025-07595-3 (PMC12624995; doi:10.1186/s12870-025-07595-3)
Supplement: Supplementary file 2 — Supplementary Material 2. [file 12870_2025_7595_MOESM2_ESM.docx]

**Figure S2**: Evolution of contrasting climatic conditions at the two experimental sites, Duclos (blue) and Godet (red). Global radiation (A), precipitation height (B), daily maximum and minimum temperatures (C), as well as daily maximum and minimum humidity levels (D) are presented for the period from 01/01/2021 to 01/08/2021. The data were collected from the Climatik database (INRA).
